# 2-Hydr­oxy-3-methoxy­benzoic acid monohydrate

**DOI:** 10.1107/S1600536808006065

**Published:** 2008-03-12

**Authors:** Zhan-Qiang Fang, Rong-Hua Zeng, Mei Yang, Hui Liu, Xiao-Lei Chen

**Affiliations:** a School of Chemistry and Environment, South China Normal University, Guangzhou 510006, People’s Republic of China

## Abstract

The asymmetric unit of the title compound, C_8_H_8_O_4_·H_2_O, contains two organic mol­ecules which are connected by the two water mol­ecules through O—H⋯O hydrogen bonds, forming an *R*_4_^4^(12) ring. Further O—H⋯O hydrogen bonds assemble these rings through *R*_6_^6^(18) rings, giving rise to infinite helical chains arranged around the *b* axis. These helical chains are assembled by offset π–π stacking inter­actions [centroid–centroid distance = 3.6432 (8) Å] between the aromatic rings of neigboring chains, forming a supra­molecular network.

## Related literature

For related literature, see: Kozlevcar *et al.* (2006 ▶); Moncol *et al.* (2006 ▶); Liu *et al.* (2007 ▶); Bernstein *et al.* (1995 ▶); Etter *et al.* (1990 ▶).
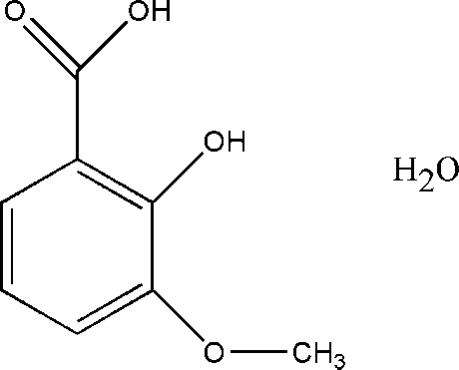


## Experimental

### 

#### Crystal data

C_8_H_8_O_4_·H_2_O
*M* *_r_* = 186.16
Monoclinic, 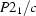

*a* = 17.9642 (4) Å
*b* = 14.5225 (3) Å
*c* = 6.8864 (2) Å
β = 91.770 (1)°
*V* = 1795.70 (8) Å^3^
*Z* = 8
Mo *K*α radiation
μ = 0.12 mm^−1^
*T* = 296 (2) K
0.30 × 0.25 × 0.20 mm

#### Data collection

Bruker APEXII area-detector diffractometer
Absorption correction: none
17337 measured reflections
4111 independent reflections
2658 reflections with *I* > 2σ(*I*)
*R*_int_ = 0.026

#### Refinement

*R*[*F* ^2^ > 2σ(*F* ^2^)] = 0.040
*wR*(*F* ^2^) = 0.110
*S* = 1.04
4111 reflections
241 parameters
H-atom parameters constrained
Δρ_max_ = 0.12 e Å^−3^
Δρ_min_ = −0.17 e Å^−3^

### 

Data collection: *APEX2* (Bruker, 2004 ▶); cell refinement: *SAINT* (Bruker, 2004 ▶); data reduction: *SAINT*; program(s) used to solve structure: *SHELXS97* (Sheldrick, 2008 ▶); program(s) used to refine structure: *SHELXL97* (Sheldrick, 2008 ▶); molecular graphics: *ORTEPIII* (Burnett & Johnson, 1996 ▶), *ORTEP-3 for Windows* (Farrugia, 1997 ▶) and *PLATON* (Spek, 2003 ▶); software used to prepare material for publication: *SHELXTL* (Sheldrick, 2008 ▶).

## Supplementary Material

Crystal structure: contains datablocks I, global. DOI: 10.1107/S1600536808006065/dn2309sup1.cif

Structure factors: contains datablocks I. DOI: 10.1107/S1600536808006065/dn2309Isup2.hkl

Additional supplementary materials:  crystallographic information; 3D view; checkCIF report

## Figures and Tables

**Table 1:** Hydrogen-bond geometry (Å, °)

*D*—H⋯*A*	*D*—H	H⋯*A*	*D*⋯*A*	*D*—H⋯*A*
O2—H2⋯O3	0.82	1.90	2.6142 (14)	144
O4—H4*A*⋯O2*W*	0.82	1.77	2.5634 (16)	164
O5—H5*A*⋯O1*W*	0.82	1.78	2.5763 (16)	165
O7—H7⋯O6	0.82	1.88	2.5946 (14)	145
O1*W*—H1*W*⋯O3	0.85	1.96	2.8082 (16)	171
O2*W*—H4*W*⋯O6	0.85	1.97	2.8071 (16)	170
O1*W*—H2*W*⋯O1^i^	0.84	2.11	2.8741 (17)	150
O1*W*—H2*W*⋯O2^i^	0.84	2.49	3.1930 (16)	141
O2*W*—H3*W*⋯O8^ii^	0.84	2.13	2.8866 (19)	149
O2*W*—H3*W*⋯O7^ii^	0.84	2.33	3.0331 (15)	141

Symmetry codes: (i) 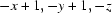; (ii) 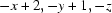.

**Fig. 1.**
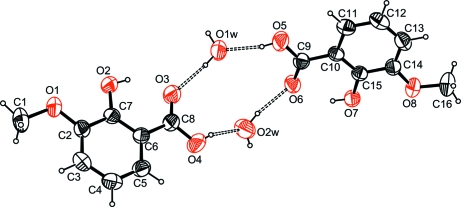
The molecular structure showing the atomic-numbering scheme. Displacement ellipsoids are drawn at the 50% probability level. H bonds are shown as dashed lines. H atoms are represented as small spheres of arbitrary radii.

**Fig. 2.**
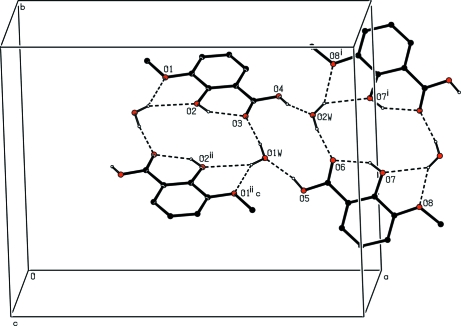
Partial packing view showing the intermolecular hydrogen bonding interactions as dashed lines. H atoms not involved in hydrogen bondings have been omitted for clarity. [Symmetry codes: (I) 1 - x, 1 - y, -z; (ii) 2 - x, 1 - y, -z]

## supplementary crystallographic information

### Comment

Hydrogen-bonding interactions between ligands are specific and directional. In this sense, 2-hydroxy-3-methoxybenzoic acid is an excellent candidate for the construction of supramolecular complexes, which have multiple coordination modes and form regular hydrogen bonds functioning as both a hydrogen-bond donor and acceptor (Kozlevcar *et al.*, 2006, Liu *et al.*, 2007, Moncol *et al.*, 2006). Recently, we obtained the title compound of (I) under hydrothermal condition.

The asymmetric unit of the title compound (I) contains two molecules which are connected by the water molecules trough O—H···O hydrogen bonds building up a *R*_4_^4^(12) ring (Etter *et al.*, 1990; Bernstein *et al.*, 1995) (Table 1, Fig. 1). The C—C and C—O distances ranging from 1.225 (2) to 1.425 (2) Å, show no remarkable features. These rings are further connected to each other by O—H···O hydrogen bonds buiding a *R*_6_^6^ (18) ring to form helical chains arranged around the [0 1/2 0] axis (Table 1, Fig. 2). These helical chains are further assembled through offset π-π stacking interactions between the aromatic rings of neigboring chains (centroid to centroid distance of 3.6432 (8) Å [Symmetry code: 1 - *x*,1 - *y*,-1 - *z*]; interplanar distance of 3.44 Å and slippest distances of 1.20 Å) to form a supramolecular network.

### Experimental

2-Hydroxy-3-methoxybenzoic acid was dissolved in hot water with stirring. Colorless single crystals suitable for X-ray diffraction were obtained at room temperature by slow evaporation of the solvent over a period of several days.

### Refinement

H atoms on 2-hydroxy-3-methoxybenzoic acid were placed at calculated positions and were treated as riding on the parent C atoms with C—H = 0.93 Å (aromatic ring) or 0.96 Å (methyl group), O—H = 0.82 Å (hydroxyl and carboxylate groups) and with *U*_iso_(H) = 1.2 or 1.5 *U*_eq_(C, O). Water H atoms were tentatively located in difference Fourier maps and were refined with distance restraints of O–H = 0.85 Å and H···H = 1.39 Å, each within a standard deviation of 0.01 Å, and with *U*_iso_(H) = 1.5 *U*_eq_(O). In the last stage of refinement, the H attached to water molecule were treated as riding on their parent O atoms.

### Crystal data

**Table:**

C_8_H_8_O_4_·H_2_O	*F*_000_ = 784
*M**_r_* = 186.16	*D*_x_ = 1.377 Mg m^−^^3^
Monoclinic, *P*2_1_/*c*	Mo *K*α radiation λ = 0.71073 Å
Hall symbol: -P 2ybc	Cell parameters from 3600 reflections
*a* = 17.9642 (4) Å	θ = 1.4–28.0º
*b* = 14.5225 (3) Å	µ = 0.12 mm^−^^1^
*c* = 6.8864 (2) Å	*T* = 296 (2) K
β = 91.770 (1)º	Block, colorless
*V* = 1795.70 (8) Å^3^	0.30 × 0.25 × 0.20 mm
*Z* = 8	

### Data collection

**Table:**

Bruker APEXII area-detector diffractometer	2658 reflections with *I* > 2σ(*I*)
Radiation source: fine-focus sealed tube	*R*_int_ = 0.026
Monochromator: graphite	θ_max_ = 27.5º
*T* = 296(2) K	θ_min_ = 1.1º
φ and ω scans	*h* = −23→23
Absorption correction: none	*k* = −18→16
17337 measured reflections	*l* = −8→8
4111 independent reflections	

### Refinement

**Table:**

Refinement on *F*^2^	Secondary atom site location: difference Fourier map
Least-squares matrix: full	Hydrogen site location: inferred from neighbouring sites
*R*[*F*^2^ > 2σ(*F*^2^)] = 0.040	H-atom parameters constrained
*wR*(*F*^2^) = 0.110	*w* = 1/[σ^2^(*F*_o_^2^) + (0.0466*P*)^2^ + 0.2399*P*] where *P* = (*F*_o_^2^ + 2*F*_c_^2^)/3
*S* = 1.04	(Δ/σ)_max_ = 0.001
4111 reflections	Δρ_max_ = 0.12 e Å^−^^3^
241 parameters	Δρ_min_ = −0.17 e Å^−^^3^
Primary atom site location: structure-invariant direct methods	Extinction correction: none

### Special details

**Table:**

Geometry. All e.s.d.'s (except the e.s.d. in the dihedral angle between two l.s. planes) are estimated using the full covariance matrix. The cell e.s.d.'s are taken into account individually in the estimation of e.s.d.'s in distances, angles and torsion angles; correlations between e.s.d.'s in cell parameters are only used when they are defined by crystal symmetry. An approximate (isotropic) treatment of cell e.s.d.'s is used for estimating e.s.d.'s involving l.s. planes.
Refinement. Refinement of *F*^2^ against ALL reflections. The weighted *R*-factor *wR* and goodness of fit *S* are based on *F*^2^, conventional *R*-factors *R* are based on *F*, with *F* set to zero for negative *F*^2^. The threshold expression of *F*^2^ > σ(*F*^2^) is used only for calculating *R*-factors(gt) *etc*. and is not relevant to the choice of reflections for refinement. *R*-factors based on *F*^2^ are statistically about twice as large as those based on *F*, and *R*- factors based on ALL data will be even larger.

### Fractional atomic coordinates and isotropic or equivalent isotropic displacement parameters (Å^2^)

**Table:**

	*x*	*y*	*z*	*U*_iso_*/*U*_eq_	
C1	0.32586 (10)	0.67219 (16)	−0.6058 (3)	0.0809 (6)	
H1A	0.3210	0.6377	−0.7244	0.121*	
H1B	0.3391	0.7346	−0.6347	0.121*	
H1C	0.2794	0.6714	−0.5406	0.121*	
C2	0.45420 (8)	0.63558 (10)	−0.5448 (2)	0.0460 (4)	
C3	0.47543 (9)	0.66626 (11)	−0.7236 (2)	0.0549 (4)	
H3	0.4396	0.6869	−0.8138	0.066*	
C4	0.54984 (9)	0.66664 (11)	−0.7702 (2)	0.0560 (4)	
H4	0.5638	0.6874	−0.8915	0.067*	
C5	0.60295 (9)	0.63668 (11)	−0.6385 (2)	0.0515 (4)	
H5	0.6528	0.6367	−0.6711	0.062*	
C6	0.58266 (8)	0.60588 (10)	−0.4548 (2)	0.0428 (3)	
C7	0.50804 (8)	0.60518 (10)	−0.4073 (2)	0.0414 (3)	
C8	0.63923 (8)	0.57528 (11)	−0.3100 (2)	0.0498 (4)	
C9	0.86646 (8)	0.35192 (11)	0.1597 (2)	0.0455 (4)	
C10	0.92233 (7)	0.27809 (10)	0.16407 (19)	0.0399 (3)	
C11	0.90153 (9)	0.18508 (10)	0.1659 (2)	0.0498 (4)	
H11	0.8514	0.1691	0.1681	0.060*	
C12	0.95461 (10)	0.11785 (11)	0.1646 (2)	0.0555 (4)	
H12	0.9404	0.0563	0.1647	0.067*	
C13	1.02955 (10)	0.14065 (12)	0.1631 (2)	0.0550 (4)	
H13	1.0653	0.0943	0.1628	0.066*	
C14	1.05131 (8)	0.23114 (11)	0.1620 (2)	0.0464 (4)	
C15	0.99746 (8)	0.30129 (10)	0.1617 (2)	0.0400 (3)	
C16	1.18105 (11)	0.19618 (16)	0.1478 (4)	0.0897 (7)	
H16A	1.1824	0.1585	0.2623	0.135*	
H16B	1.2279	0.2272	0.1366	0.135*	
H16C	1.1720	0.1582	0.0354	0.135*	
O1	0.38246 (5)	0.63195 (9)	−0.48365 (16)	0.0625 (3)	
O2	0.48284 (5)	0.57751 (8)	−0.23382 (15)	0.0536 (3)	
H2	0.5180	0.5596	−0.1648	0.080*	
O3	0.62337 (6)	0.54527 (9)	−0.15029 (17)	0.0642 (3)	
O4	0.70797 (6)	0.58220 (11)	−0.36507 (19)	0.0769 (4)	
H4A	0.7365	0.5686	−0.2742	0.115*	
O5	0.79730 (6)	0.32376 (8)	0.1628 (2)	0.0676 (4)	
H5A	0.7694	0.3684	0.1624	0.101*	
O6	0.88288 (6)	0.43402 (8)	0.15148 (17)	0.0549 (3)	
O7	1.02269 (5)	0.38892 (7)	0.15833 (16)	0.0502 (3)	
H7	0.9872	0.4245	0.1510	0.075*	
O8	1.12298 (6)	0.26247 (8)	0.16093 (18)	0.0635 (3)	
O1W	0.69046 (7)	0.44218 (10)	0.1525 (2)	0.0860 (5)	
H1W	0.6738	0.4780	0.0633	0.129*	
H2W	0.6557	0.4307	0.2287	0.129*	
O2W	0.81581 (7)	0.55507 (10)	−0.1193 (2)	0.0943 (5)	
H3W	0.8486	0.5957	−0.1336	0.141*	
H4W	0.8309	0.5188	−0.0299	0.141*	

### Atomic displacement parameters (Å^2^)

**Table:**

	*U*^11^	*U*^22^	*U*^33^	*U*^12^	*U*^13^	*U*^23^
C1	0.0531 (11)	0.1004 (17)	0.0885 (14)	0.0254 (10)	−0.0105 (9)	0.0183 (12)
C2	0.0432 (8)	0.0408 (9)	0.0540 (9)	0.0038 (7)	0.0006 (7)	0.0023 (7)
C3	0.0601 (10)	0.0499 (10)	0.0543 (10)	0.0075 (8)	−0.0055 (8)	0.0082 (8)
C4	0.0676 (11)	0.0505 (10)	0.0502 (9)	−0.0019 (8)	0.0068 (8)	0.0091 (7)
C5	0.0490 (9)	0.0484 (10)	0.0575 (10)	−0.0037 (7)	0.0098 (7)	0.0030 (7)
C6	0.0417 (8)	0.0371 (8)	0.0497 (8)	−0.0026 (6)	0.0019 (6)	0.0015 (6)
C7	0.0440 (8)	0.0338 (8)	0.0464 (8)	−0.0013 (6)	0.0027 (6)	0.0009 (6)
C8	0.0412 (8)	0.0511 (10)	0.0570 (10)	−0.0026 (7)	0.0017 (7)	0.0022 (8)
C9	0.0425 (8)	0.0477 (10)	0.0460 (8)	−0.0029 (7)	−0.0014 (6)	0.0013 (7)
C10	0.0445 (8)	0.0391 (8)	0.0360 (7)	−0.0014 (6)	−0.0006 (6)	0.0010 (6)
C11	0.0559 (9)	0.0446 (10)	0.0486 (9)	−0.0072 (7)	−0.0033 (7)	0.0038 (7)
C12	0.0756 (12)	0.0376 (9)	0.0529 (9)	−0.0016 (8)	−0.0031 (8)	0.0041 (7)
C13	0.0683 (11)	0.0469 (10)	0.0498 (9)	0.0154 (8)	0.0030 (8)	0.0028 (7)
C14	0.0469 (8)	0.0511 (10)	0.0411 (8)	0.0069 (7)	0.0031 (6)	0.0021 (7)
C15	0.0451 (8)	0.0403 (9)	0.0346 (7)	−0.0007 (6)	0.0007 (6)	0.0004 (6)
C16	0.0574 (12)	0.0963 (18)	0.1162 (18)	0.0313 (11)	0.0147 (11)	0.0095 (13)
O1	0.0400 (6)	0.0788 (9)	0.0686 (7)	0.0130 (5)	−0.0003 (5)	0.0157 (6)
O2	0.0429 (6)	0.0693 (8)	0.0487 (6)	0.0036 (5)	0.0049 (5)	0.0124 (5)
O3	0.0484 (6)	0.0880 (9)	0.0562 (7)	0.0054 (6)	−0.0007 (5)	0.0164 (6)
O4	0.0377 (6)	0.1133 (11)	0.0795 (9)	−0.0058 (7)	0.0002 (6)	0.0279 (8)
O5	0.0390 (6)	0.0566 (8)	0.1071 (10)	−0.0032 (5)	−0.0005 (6)	0.0053 (7)
O6	0.0484 (6)	0.0409 (7)	0.0751 (8)	0.0013 (5)	−0.0012 (5)	0.0016 (5)
O7	0.0420 (6)	0.0414 (7)	0.0670 (7)	−0.0031 (5)	0.0010 (5)	−0.0005 (5)
O8	0.0418 (6)	0.0673 (8)	0.0816 (8)	0.0115 (5)	0.0084 (5)	0.0044 (6)
O1W	0.0604 (8)	0.1133 (12)	0.0857 (9)	0.0343 (7)	0.0227 (7)	0.0390 (8)
O2W	0.0522 (7)	0.0961 (11)	0.1329 (13)	−0.0188 (7)	−0.0272 (8)	0.0534 (9)

### Geometric parameters (Å, °)

**Table:**

C1—O1	1.4247 (19)	C10—C11	1.402 (2)
C1—H1A	0.9600	C11—C12	1.365 (2)
C1—H1B	0.9600	C11—H11	0.9300
C1—H1C	0.9600	C12—C13	1.387 (2)
C2—O1	1.3692 (17)	C12—H12	0.9300
C2—C3	1.374 (2)	C13—C14	1.371 (2)
C2—C7	1.4036 (19)	C13—H13	0.9300
C3—C4	1.384 (2)	C14—O8	1.3657 (18)
C3—H3	0.9300	C14—C15	1.405 (2)
C4—C5	1.367 (2)	C15—O7	1.3514 (17)
C4—H4	0.9300	C16—O8	1.425 (2)
C5—C6	1.401 (2)	C16—H16A	0.9600
C5—H5	0.9300	C16—H16B	0.9600
C6—C7	1.3895 (19)	C16—H16C	0.9600
C6—C8	1.470 (2)	O2—H2	0.8200
C7—O2	1.3520 (17)	O4—H4A	0.8200
C8—O3	1.2250 (19)	O5—H5A	0.8200
C8—O4	1.3067 (18)	O7—H7	0.8200
C9—O6	1.2299 (18)	O1W—H1W	0.8516
C9—O5	1.3088 (17)	O1W—H2W	0.8440
C9—C10	1.468 (2)	O2W—H3W	0.8422
C10—C15	1.3916 (19)	O2W—H4W	0.8477
			
O1—C1—H1A	109.5	C15—C10—C9	119.05 (13)
O1—C1—H1B	109.5	C11—C10—C9	121.44 (13)
H1A—C1—H1B	109.5	C12—C11—C10	120.19 (15)
O1—C1—H1C	109.5	C12—C11—H11	119.9
H1A—C1—H1C	109.5	C10—C11—H11	119.9
H1B—C1—H1C	109.5	C11—C12—C13	120.51 (15)
O1—C2—C3	125.31 (13)	C11—C12—H12	119.7
O1—C2—C7	114.58 (13)	C13—C12—H12	119.7
C3—C2—C7	120.11 (14)	C14—C13—C12	120.39 (15)
C2—C3—C4	120.36 (14)	C14—C13—H13	119.8
C2—C3—H3	119.8	C12—C13—H13	119.8
C4—C3—H3	119.8	O8—C14—C13	126.03 (14)
C5—C4—C3	120.29 (15)	O8—C14—C15	114.06 (14)
C5—C4—H4	119.9	C13—C14—C15	119.91 (14)
C3—C4—H4	119.9	O7—C15—C10	123.65 (13)
C4—C5—C6	120.33 (15)	O7—C15—C14	116.84 (13)
C4—C5—H5	119.8	C10—C15—C14	119.50 (13)
C6—C5—H5	119.8	O8—C16—H16A	109.5
C7—C6—C5	119.62 (13)	O8—C16—H16B	109.5
C7—C6—C8	119.36 (13)	H16A—C16—H16B	109.5
C5—C6—C8	121.01 (13)	O8—C16—H16C	109.5
O2—C7—C6	124.17 (12)	H16A—C16—H16C	109.5
O2—C7—C2	116.55 (13)	H16B—C16—H16C	109.5
C6—C7—C2	119.27 (14)	C2—O1—C1	117.63 (13)
O3—C8—O4	122.41 (14)	C7—O2—H2	109.5
O3—C8—C6	122.77 (14)	C8—O4—H4A	109.5
O4—C8—C6	114.82 (14)	C9—O5—H5A	109.5
O6—C9—O5	122.19 (14)	C15—O7—H7	109.5
O6—C9—C10	122.96 (13)	C14—O8—C16	117.88 (15)
O5—C9—C10	114.85 (14)	H1W—O1W—H2W	108.6
C15—C10—C11	119.49 (13)	H3W—O2W—H4W	108.1

### Hydrogen-bond geometry (Å, °)

**Table:**

*D*—H···*A*	*D*—H	H···*A*	*D*···*A*	*D*—H···*A*
O2—H2···O3	0.82	1.90	2.6142 (14)	144
O4—H4A···O2W	0.82	1.77	2.5634 (16)	164
O5—H5A···O1W	0.82	1.78	2.5763 (16)	165
O7—H7···O6	0.82	1.88	2.5946 (14)	145
O1W—H1W···O3	0.85	1.96	2.8082 (16)	171
O2W—H4W···O6	0.85	1.97	2.8071 (16)	170
O1W—H2W···O1^i^	0.84	2.11	2.8741 (17)	150
O1W—H2W···O2^i^	0.84	2.49	3.1930 (16)	141
O2W—H3W···O8^ii^	0.84	2.13	2.8866 (19)	149
O2W—H3W···O7^ii^	0.84	2.33	3.0331 (15)	141

Symmetry codes: (i) −*x*+1, −*y*+1, −*z*; (ii) −*x*+2, −*y*+1, −*z*.
